# Development of a Computerized Adaptive Assessment and Learning System for Mathematical Ability Based on Cognitive Diagnosis

**DOI:** 10.3390/jintelligence13090114

**Published:** 2025-09-02

**Authors:** Yi Zhang, Liping Zhang, Heyang Zhang, Xiaopeng Wu

**Affiliations:** 1 School of Education, Guangzhou University, Guangzhou 510006, China; yzhang@gzhu.edu.cn; 2 Faculty of Education, Northeast Normal University, Changchun 130024, China; zhanglp125@nenu.edu.cn; 3 School of Mathematics and Statistics, Northeast Normal University, Changchun 130024, China; zhanghy334@nenu.edu.cn

**Keywords:** adaptive learning system, cognitive diagnosis assessment, online learning

## Abstract

With the rapid evolution of technology and the continuous deepening of digital transformation in education, personalized and adaptive learning have emerged as inevitable trends in the educational landscape. This study focuses on a Computerized Adaptive Learning System Based on Cognitive Diagnosis (CAL-CDS)—an integrated platform that incorporates multiple technologies for assessment and learning. The study is organized around two dimensions: (1) constructing a foundational cognitive diagnostic assessment framework, and (2) investigating the operational mechanisms of the cognitive diagnosis-based computerized adaptive system. It comprehensively incorporates core components including cognitive modeling, Q-matrix generation, and diagnostic test development. On this basis, this study dissects the system’s operational logic from four aspects: the adaptive testing system, diagnostic system, recommendation system, and empirical case studies. This study effectively addresses two core questions: how to construct a cognitive diagnostic assessment framework that alignes with China’s mathematics knowledge structure, and how to facilitate personalized student learning via cognitive diagnosis. Overall, this study offers a systematic solution for developing mathematics-specific cognitive diagnosis-driven adaptive learning systems.

## 1. Introduction

The proliferation of information and communication technologies (ICTS) has facilitated the widespread adoption of online learning platforms, thereby providing unprecedented flexibility for learners in different educational settings (Aboagye et al. 2020). However, traditional online learning systems typically employ a “one-size-fits-all” approach (Colchester et al. 2017), which fails to account for individual differences in cognitive abilities, knowledge states, and learning trajectories. Consequently, this limitation exacerbates problems of learner disengagement, inefficient knowledge acquisition and inadequate personalized support (Kibuku et al. 2020). In response to this challenge, adaptive learning systems have emerged as a promising solution that leverage student data, learning process data, and learning outcome data to dynamically adapt instructional content and activities to individual abilities or learning preferences, thereby delivering efficient, effective, and customized learning experiences. (Khosravi et al. 2020). Multiple studies indicate that adaptive learning systems exert a positive impact on students’ academic performance (Xie et al. 2019; Vanlehn 2011).

In recent years, research on adaptive learning tools (systems) related to mathematical skills, particularly those incorporating diagnostic assessment capabilities, has demonstrated significant advancements, with diverse developments observed both domestically and internationally. Internationally, assessment tools for mathematical skills, grounded in cognitive diagnostic theory, primarily focus on evaluating students’ mastery of fundamental skills (such as algebra, geometry, arithmetic, etc.). Their core models include DINA, G-DINA, and R-RUM, among others. Representative systems, like ASSISTments (Heffernan and Heffernan 2014) in the United States, have implemented real-time feedback functionalities based on cognitive diagnostic models in sub-domains such as algebra and functions, and are widely applied in mathematics learning analytics for K-12 education. Meanwhile, researchers have also explored integrated modeling approaches that integrate Cognitive Diagnostic Models (CDM) with Item Response Theory (IRT)**,** aiming to further enhance the precision and adaptability of diagnostic assessments. Liu et al. (2015) proposed a Cognitive Diagnostic Computerized Adaptive Testing (CD-CAT) model based on rate functions, achieving a balance between diagnostic accuracy and testing efficiency. The Aplusix system developed in France employs formal mathematical rules to construct a reasoning path validation model, with its core mechanism involving the analysis of intermediate steps in students’ mathematical problem-solving processes to enable real-time diagnosis of algebraic errors (Chaachoua et al. 2004; Nicaud et al. 2004). Additionally, research, along with secondary development efforts based on PISA and TIMSS data, has demonstrated strong diagnostic efficacy. However, it is crucial to note that while cognitive diagnostic models exhibit high theoretical diagnostic validity in research contexts, their practical implementation in Chinese classroom settings still faces substantial challenges. Prior research has confirmed that the effectiveness of these models largly depends on the alignment between the Q-matrix and attribute definitions and the local curriculum standards (Rupp and Templin 2008). Furthermore, scholars have pointed out that foreign attribute models, which have not undergone local validation, are often incompatible with China’s instructional objectives and item structures, thereby directly undermining the validity of diagnostic conclusions (Leighton and Gierl 2007a). In China, the Taiwan region has developed the “Adaptive Learning Network” (Yin Cai Wang) platform based on CD-CAT technology, covering three subjects: Chinese Language, Mathematics, and Science. Chinese Language and Mathematics span 9 grade levels, while Science covers 6 grade levels. It includes over 3000 knowledge points, more than 20,000 diagnostic items, over 150 interactive tutoring modules, and more than 1000 dynamic assessments. It has established an auxiliary learning platform for elementary education suitable for students in the Taiwan region (Zhu and Zhang 2021). Furthermore, in recent years, assessment platforms have integrated Cognitive Diagnostic Testing (CDT). CDT-integrated assessment platforms represented by “precision learning” and “Smart Classroom” have gradually emerged. While these platforms demonstrate significant systematic advantages, the alignment of their theoretical models with international standards requires further strengthening to achieve standardization. Additionally, some platforms exhibit a technology-first orientation in their development process, failing to ground themselves in cognitive science as their core theoretical foundation (Chen et al. 2021). Against this backdrop, developing cognitive diagnostic assessment tools that are highly compatible with China’s mathematics curriculum system has become a crucial initiative for enhancing the quality of precision teaching. Therefore, building upon existing CDT tools, this study not only proposes adaptive adjustment strategies by integrating local curriculum content, curriculum standards, and cognitive characteristics, but also constructs a compatible Q-matrix and diagnostic model, aiming to realize a computerized diagnostic assessment system for mathematical skills with enhanced practical applicability.

Cognitive Diagnostic Assessment (CDA) is a theoretical basis for personalizing learning with adaptive learning systems (Wu et al. 2022). It aims to infer learners’ mastery of cognitive skills through the use of cognitive science and statistical modeling, further constructing fine-grained reports and providing formative feedback (DiBello et al. 2012; Nichols 1994; Templin and Henson 2006). That is, in cognitive diagnostic assessment, based on the characteristics of psychological assessment, detailed information on students’ diagnosis can be obtained through the analysis of items with cognitive diagnostic functions and mathematical models, thereby enabling personalized assessment and facilitating adaptive learning (Henson et al. 2009; Rupp and Templin 2008). Cognitive diagnostic assessment presents tasks to examinees in the form of items, utilizing their response outcomes as diagnostic data. Concurrently, respondents’ latent traits are defined as the requisite “attributes” for diagnosis, which are subsequently operationalized into quantifiable latent variables. Ultimately, we analyze the interrelationship between these variables and data via psychometric models, thereby forming a complete CDA workflow (Templin and Henson 2010). And cognitive attributes and cognitive models are the core concepts that make up CDA (Wu et al. 2020). Cognitive attributes refer to the individual knowledge and skills that the assessment focuses on (Gierl 2007), namely the mathematical competencies or content knowledge required to solve test items (Choi et al. 2015), such as the rules applied by students in rational number subtraction (Tatsuoka 1983). Broadly speaking, students engage with content knowledge, cognitive processes, and procedural skills when undertaking problem-solving tasks (Dogan and Tatsuoka 2008; Leighton et al. 2004; Leighton and Gierl 2007b). Researchers have argued that hierarchically organized cognitive attributes can illuminate the mental order between the attributes required to solve a test problem, and that test takers can only acquire subsequent attributes if they possess the antecedent attributes (Leighton et al. 2004). The structure of cognitive attributes, namely cognitive models, is defined as “simplified descriptions of human problem-solving processes in standardized educational tasks which serves to characterize the knowledge and skills acquired by students at different levels of learning and facilitate the interpretation and prediction of student performance” (Leighton and Gierl 2007b, p. 6). From a statistical perspective, cognitive diagnostic models (CDMs) represent an extension of confirmatory latent class psychometric models (Von Davier 2008). In most multidimensional item response theory (MIRT) and confirmatory factor analysis (CFA) applications, the analyst aims to construct what can be informally termed a simple loading structure, where each item loads on only one dimension. In contrast, cognitive models can fully realize their theoretical potential, particularly within complex loading structures where each item loads on multiple dimensions. CDMs provide stakeholders with feedback about a learner’s strengths and weaknesses based on their mastery or non-mastery of specific cognitive attributes. This contrasts with Classical Test Theory (CTT) and IRT frameworks, which typically report only a single psychometric measure (such as a total score) relative to a reference group (Crocker and Algina 1986). The core task of cognitive diagnosis is to uncover the intrinsic relationships among item characteristics, examinee traits, and response patterns. By analyzing test-takers’ responses an examinee’s responses, it infers which attributes have been mastered and which have not, thereby enabling insights into learning processes, exploration of effective learning paths, implementation of remedial instruction to achieve instructional objectives. Essentially, CDM constitutes a multidimensional latent variable classification framework, which has garnered considerable favor within the measurement community due to their strong capacity for analyzing fine-grained skills. These skills frequently interact, collectively affecting a learner’s responses to test items (Rupp and Templin 2008). Conversely, most models developed within the CTT and IRT frameworks are unidimensional, producing total scores that often merely reflect an overall ability in the general domain assessed by the test (Crocker and Algina 1986). Based on cognitive modeling, CDA applies cognitive science and statistical modeling to infer students’ mastery of their cognitive skills, generate detailed reports, and provide meaningful feedback (Nichols 1994). In this way, CDA-based adaptive learning systems enable personalized learning by detecting students deficiencies through human–computer interaction and delivering targeted instructional interventions (Zhang et al. 2024). Building on the foundation of previously developed learning systems that achieved preliminary success in personalized education, this study innovatively uses cognitive diagnostic theory to develop a student-centered adaptive learning system that combines test diagnosis and management. This paper provides an overview of this cognitive diagnostic-based adaptive learning system and shares reflections and lessons from its development and pilot implementation. While the research primarily focuses on adaptive learning system development, many challenges encountered are applicable to broader educational tools and technologies. The purpose of the research is to offer references to other scholars intending to adopt adaptive learning systems or engage in the implementation of educational tools and technologies. Additionlly, in line with the research needs, this paper puts forward the following two research questions:

RQ1: How can we construct a cognitive diagnostic model that aligns with the characteristics of Chinese mathematics knowledge structures?

RQ2: How can we implement personalized student learning based on cognitive diagnostic assessment?

The remaining sections of this paper are structured as follows: Section 2 describes the preliminaries of developing the system including the content selected, the ISM methodology, and the expert methodology, as well as the development process involving Q-matrix construction and assessment tool design; Section 3 details the results of this study, that is, the architecture and operational processes of the developed adaptive learning system; Section 4 discusses the present study; and Section 5 addresses the limitations of this study and future directions.

## 2. Cognitive Diagnostic Assessment Foundation System Construction

### 2.1. Selection of Knowledge Modules

Given that the research aims to develop an online adaptive learning system tailored for Chinese high school students, the selection of knowledge domains must align with the actual Chinese high school mathematics curriculum. This study references the latest Chinese High School Mathematics Curriculum Standards (2017 Edition) (MOE 2018) and preliminarily selects four universal themes in the compulsory senior high school courses—Preparatory Knowledge, Functions, Geometry and Algebra, and Probability and Statistics—as well as three themes in the optional compulsory courses—Functions, Geometry and Algebra, and Probability and Statistics. In order to further identify specific knowledge modules, this study employed the method of literature analysis to make a careful comb of the literature on the modular analysis of the college entrance examination mathematics questions in recent years (in China, the college entrance examination is of vital importance, and reference to the college entrance examination mathematics examination papers is of great value). Based on a literature analysis, it was found that National College Entrance Examination (Gaokao) mathematics questions in thte last five years emphasize foundational knowledge areas such as functions, equations, sequences, solid geometry, and plane analytic geometry (He 2025; Liu 2025; Liu et al. 2025). Accordingly, ten modules are identified at this stage: “Concepts and Properties of Functions,” “Basic Properties of Trigonometric Functions,” “Basic Solid Geometry,” “Space Vectors and Solid Geometry,” “Equations and Inequalities,” “Circles and Equations,” “Lines and Equations,” “Probability and Independence of Random Events,” “Estimating Population Parameters from Samples,” and “Sampling.” After conducting three rounds of consultation with three mathematics education researchers, five teacher researchers, and seven special teachers in the field, it was decided to exclude the module “Sampling” and add “Discrete Random Variables and Normal Distributions” and “Linear Regression and Independent Testing”. The final 11 knowledge modules are listed in Table 1.

### 2.2. Construction of Cognitive Models

**Figure 1:**
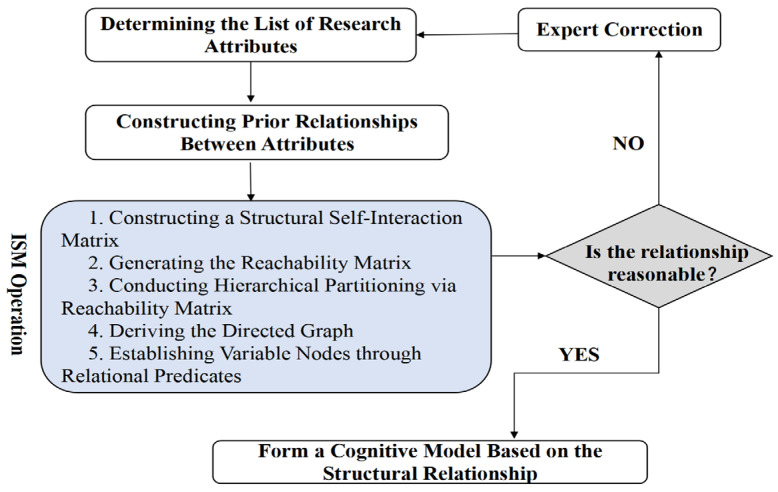
The process of interpretive structural modeling.

After identifing 11 knowledge modules, the study focus shifted to developing appropriate cognitive models for each module. In educational research, cognitive modeling is typically defined as “the cognitive processes that are used to describe students’ problem-solving on tests” (Leighton and Gierl 2007a). These models not only help identify knowledge and skills corresponding to students’ different learning levels, but also offer explanations and predictions regarding student performance (Leighton and Gierl 2007a). High-quality cognitive modeling requires collaboration among teaching professionals, psychologists, measurement scientists, and teachers. However, there are no well-established methods for developing cognitive models (Sun et al. 2023). Current approaches predominantly rely on the delphi method, student oral reports and literature reviews—methods that suffer from low consistency and limited operability (Gierl et al. 2010). In this study, following the identification of knowledge modules, the next step is to analyze the logical relationships of these modules to construct a cognitive model. Given that the knowledge modules involve numerous complex elements, conventional modeling methods are poorly adaptable and have limited practical value. To address this complexity, this study adopts the interpretive structural modeling (ISM) method. The ISM method enables the construction of a comprehensive system model that integrates multiple components (whether directly or indirectly associated), thereby accurately characterizing the internal logic of complex situations (Attri et al. 2013a; Raj et al. 2008). The specific steps for modeling using ISM are shown in Figure 1.

**Figure 2:**
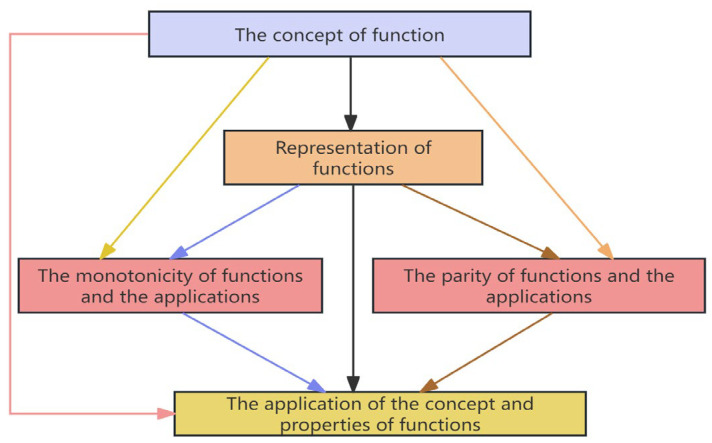
Initial directed graph.

As shown in Figure 2, the first step of modeling using ISM is to identify the attributes, which in this study refer to the knowledge points or skills contained in each module. After previously identifing 11 knowledge modules, this step will further confirm the corresponding knowledge points involved in each module according to the High School Mathematics Curriculum Standards (2017 edition) (MOE 2018). The following section briefly outlines the modeling process, taking the “Concepts and Properties of Functions” module as an example, the specific attributes of this module are presented in Table 2.

Following the determination of attributes for the module “Concepts and Properties of Functions,” the subsequent step involves clarifying the contextual relationships between each pair of attributes with the objective of identifying relationships of the “causes” or “influences” type among these attributes. Although related research primarily employs methods such as brainstorming, the Nominal Group Technique (NGT), and Creative Engineering to identify inter-attribute relationships (Ravi et al. 2005; Talib et al. 2011). This study primarily conducts questionnaires among experts in the field of mathematics education to accomplish the relationship clarification process. Once relationships are determined, a Structural Self-Interaction Matrix (SSIM) is first constructed to visualize the relationships among these attributes (Attri et al. 2013a). Subsequently, by applying computational procedures such as Boolean algebra algorithms to the SSIM, a Reachability Matrix (RM) (Yadav and Barve 2015) can be derived. Starting from the Reachability Matrix, each element can be assigned a hierarchical level based on a set of computational procedures (Yadav and Barve 2015). Top-level elements are positioned at the apex of the directed graph, followed by second-level elements below them, and so forth. All elements are interconnected according to their hierarchical levels, forming an initial directed graph composed of nodes and edges (Attri et al. 2013b). The final directed graph, which is obtained after eliminating indirect connections, serves as a visual representation of the elements and their interdependencies (Ravi and Shankar 2005). This process is illustrated in Figure 2.

**Figure 3:**
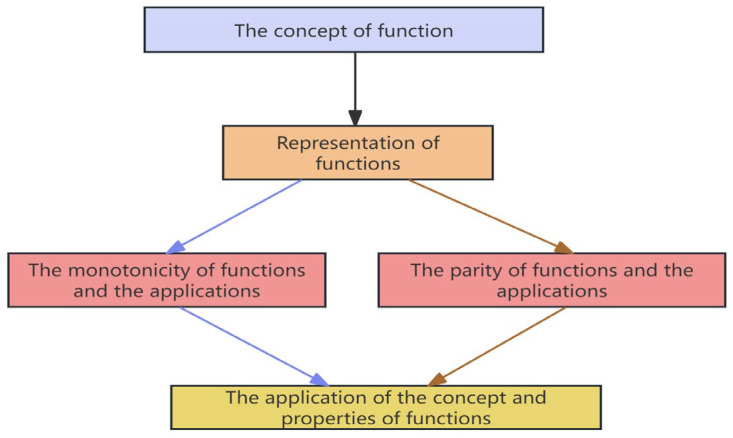
The final cognitive model.

In this study, directed graphs were employed to represent the cognitive model of a given knowledge module. The detailed steps can be found in (Zhang et al. 2024). Employing interpretive structural modeling (ISM) to construct cognitive models combines objectivity and efficiency, not only clearly elucidating the intrinsic features of knowledge domains but also aligning closely with the inherent logic of the discipline. After constructing a preliminary cognitive model of the module “Concepts and Properties of Functions” using ISM, it is necessary to revise the model using the expert method. In this model revision of all the modules, six professionals in mathematics education were invited to participate in a 2-h open seminar, where they provided modification recommendations. The paper used the revision of the cognitive model for the module “Concepts and Properties of Functions” as an example to elaborate on the method. Based on the preliminary directed graph and expert recommendations, it was identified that the influence of “the concept of function” on “monotonicity and application” and “parity and application”, respectively, the influence relationship between “function representation” and “application of function concept and properties,” and the influence relationship between “function concept” and “application of function concept and properties” are not clearly reflected in mathematics. Consequently, the four directional edges mentioned were removed from the model, resulting in the finalized cognitive model for this module, as shown in Figure 3.

### 2.3. Developing the Q-Matrices

The Q-matrix was constructed using the methods outlined in the Rule-Space Model (RSM), based on the developed cognitive models. First, the adjacency matrix was derived from the 11 previously constructed cognitive models. Subsequently, Boolean algebra methods were used to generate a preliminary Q matrix (Tatsuoka 2009). For example, taking the module “Concepts and Properties of Functions” as an example, the adjacency matrix was deduced based on the internal relationship of its cognitive model, as shown in Table 3.

Based on the adjacency matrix obtained, a Q matrix was developed to clarify the exam pattern. Considering the requirement of the number of tasks that students should complete in the exam, this study designed 2–3 items for the measurement of each attribute (Wu et al. 2022), Each item is marked in the Q-matrix with “1” or “0” in the corresponding knowledge attribute column (where “1” indicates that the item measures the attribute, and “0” indicates irrelevance). Furthermore, three independent teams were formed to conduct Q-matrix calibration tasks, with each team consisting of one educational measurement expert and one creativity researcher. To quantitatively validate the Q-matrix effectiveness, internal consistency analysis was performed: the Fleiss’ Kappa (Landis and Koch 1977) coefficient among six experts reached 0.89, which falls into the “almost perfect agreement” category. This result confirmed the validity of the constructed Q-matrix. Consequently, the finalized Q-matrix was determined. Taking the module “Concepts and Properties of Functions” as an example, the final Q-matrix is shown in Table 4.

### 2.4. Developing Assessment Tools

**Figure 4:**
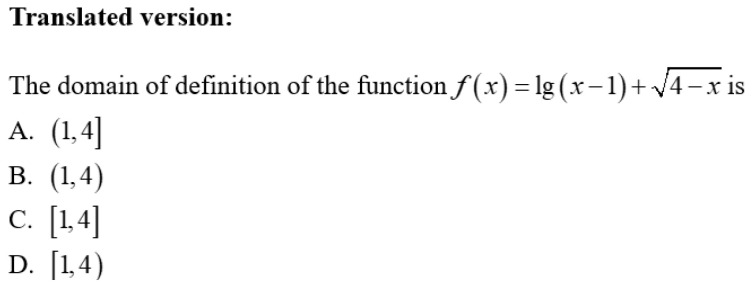
Example item for attribute *The concept of function* (**A1**).

**Figure 5:**
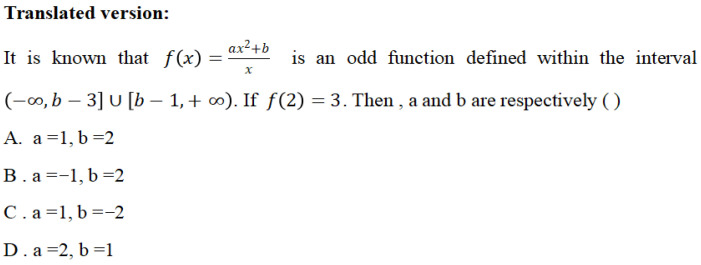
Example item for attribute The application of the concept and properties of functions (**A5**).

After the above process, the subsequent step involves selecting test items that meet the requirements of corresponding knowledge attributes based on the finalized Q-matrix. The test items used in this study were sourced from three categories: 50% were textbook exercises aligned with curriculum standards, 30% were selected from previous Gaokao (National College Entrance Examination) mathematics multiple-choice questions, and 20% were original items developed for this study. All items were appropriately adapted to meet specific research requirements. In the item design process, this study established a proposition team composed of five high school mathematics teachers to develop the test items. Additionally, the study strictly adhered to the item-attribute mapping relationship defined in the Q matrix—each multiple-choice or fill-in-the-blank question corresponded exclusively to one specific knowledge attribute in the Q matrix. For example, for attribute A1 of module “Concepts and Properties of Functions”, the corresponding test item was designed as shown in Figure 4—this item exciusively assessed students’ mastery of the concept of function. In addition, drawning on Bloom’s Taxonomy of Educational Objectives, the design of the test items for each module in this study also incorporated consideration for the division of cognitive levels. Specifically, items were categorized into three levels: memory, comprehension, and application. The memory level assessed students’ ability to recall basic concepts or formulas; the comprehension level evaluated their understanding of knowledge and capacity for simple application; and the application level measured their ability to integrate knowledge to solve complex problems (as shown in Figure 5, where students are required to comprehensively apply function knowledge to solve problems). All items were formatted as four-option multiple-choice questions, and this design approach was consistently applied to the remaining 10 knowledge modules.

After the initial design of test items for each module was completed, to further ensure the scientificity and validity of the item design, we invited five teachers with more than 10 years of teaching experience in the high school mathematics neighborhood and two experts in the field of cognitive diagnosis to examine the quality of the test items in the form of a panel of reviewers. Their evaluation focused primarily on the alignment between the topics test items and the target attributes, the reasonableness of the setting of the options and the accuracy of item wording. After three rounds of revisions in total, items with missing, redundant, or contentious content were deleted or added, linguistic expressions of individual items were adjusted for clarity, and the revised outcomes were subsequently reviewed by a review panel, ultimately achieving unanimous approval. Regarding data-driven validation, we systematically evaluated the reliability and goodness-of-fit of the diagnostic model for the identified 11 mathematical skill modules. Over 80% of the test items exhibited a good model-data fit, which indicates that the parameter estimates for the vast majority of items are reliable and that these items effectively distinguish between mastery states of their corresponding skill modules. In addition, the overall model fit was satisfactory. Specifically, with the exception of the “Probability and Independence of Random Events” module, where the indices slightly exceeded the recommended threshold, the key fit indices for the remaining 10 modules all met the established criteria (SRMSR ≤ 0.05). This indicates that the model effectively captured the response patterns of students for the vast majority of the modules. This study also calculated test–retest attribute consistency. Following the method proposed by Templin et al., which assumes that the probability of a test-taker mastering an attribute remains constant, this index was derived by calculating the correlation between the attribute mastery probabilities of the same individuals across two separate measurements (Templin and Bradshaw 2013). The results showed that the indices for nearly all skill modules exceeded 0.75 (ranging from 0.76 to 0.88). As Templin and Henson suggested, an H-index >0.70 (Templin and Henson 2006) is generally considered acceptable. This demonstrates that the diagnostic model has good and sufficient psychometric reliability for identifying students’ mastery states across the 11 mathematical skill modules, essentially meeting diagnostic requirements. Consequently, the test items (i.e., the assessment instrument) for the 11 modules in this study were finalized.

## 3. Operation Mechanism of Computerized Adaptive Learning System Based on Cognitive Diagnosis

**Figure 6:**
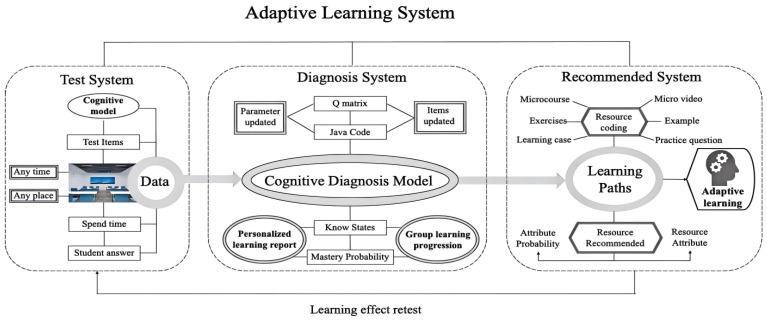
Computerized adaptive learning system based on cognitive diagnosis.

After completing the construction of the basic system of cognitive diagnostic assessment in Section 2—namely, an integrated cognitive model, a validated Q-matrix, and a comprehensive set of assessment items—these foundational materials were integrated into a computerized assessment system to enable the adaptive function. The system comprises three main components: the test system, Diagnosis System and Recommended System. Their operational mechanism are shown in Figure 6.

### 3.1. Test System

Testing is a key method for educational evaluation and a fundamental processes in personalized learning. To achieve targeted, personalized learning, it is essential to understand the knowledge status of each individual. Among various methods, testing has emerged as the most direct and effective way to do so.

As shown in Figure 6, the test system serves as the core component of the computerized adaptive learning system. Cognitive diagnostic techniques constitute the key features of this assessment. The test items in this test system are endowed with cognitive diagnostic functions. Specifically, the test construction process was grounded in a cognitive model (attributes and their hierarchical relationships), whose construction method and examples of this model are systematically described in Section 2.2. Building on the cognitive model, we established an ideal measurement model and developed a corresponding Q-matrix, as described in Section 2.3. Based on this, we constructed test items that met the cognitive diagnostic characteristics, with the item development process described in Section 2.4.

The system features two core interfaces: a student interface and an administrator (teacher) interface. The administrator interface facilitates the management of basic test item information, such as the input module supports attribute entry, test item entry, and the mapping of test items to their corresponding assessment attributes. Additionally, the examination management module enables two critical operations: generating unique student IDs and passwords for test-takers, and exporting students’ basic information after testing. Students can log in via the student interface to complete their responses to the test item.

During the testing phase, according to the specific measurement model (Q matrix), we developed multiple-context test items for each measurement model. Upon students logging in to the testing interface, the system generated a set of test items randomly based on the examination content and the measurement model (Q-matrix) for the student to answer. This approach ensured measurement consistency while allowing different tests to use distinct test items, yet achieving equivalent testing and diagnostic outcomes. At the end of the test, the system recorded the test results of each individual and transmitted the data to the diagnostic system.

### 3.2. Diagnosis System

The Diagnosis System is the core of the entire adaptive learning system, as the goal of adaptive personalized learning can be achieved only through accurate and effective diagnosis. Moreover, a cognitive diagnostic model was used for the diagnostic system. updated and iterative, we primarily utilize the Generalized Deterministic Input, Noisy “And” gate (G-DINA) model. The model is a saturated full model (De la Torre 2011). The R language package was used to encapsulate this system. Since the cognitive diagnostic model adopted by the system is a parametric model, the accuracy of the diagnosis is directly related to parameter evaluation. Notably, however, a minimum of 500 samples is required to conduct the cognitive diagnostic assessment. Therefore, during the specific implementation of the testing process, we selected 11 project schools to administer assessments for the 11 modules within the stipulated timeframes. We first initialized the model using foundational data: this involved rigorously testing each item with a sample of over 500 students to establish its parameters. The response data from these students were stored in the system as the foundational dataset. Subsequently, responses from new students taking the assessment were combined with this foundational data for joint calibration. This approach not only ensured an adequate sample size for the assessment but also facilitated effective model fitting.

**Figure 7:**
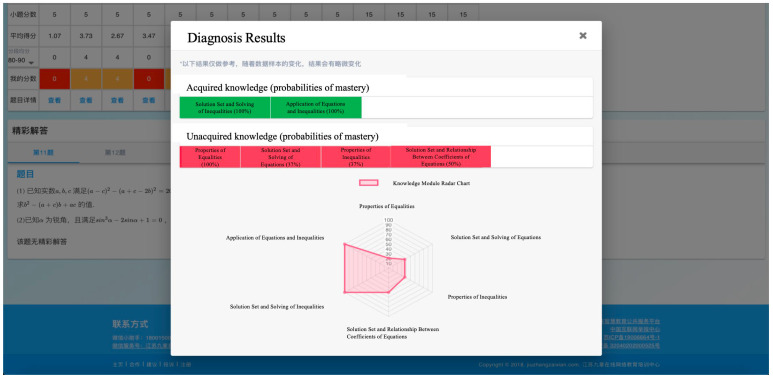
Adaptive learning system diagnostic interface.

The diagnostic system is characterized by continuous iterative updating; after a period of testing, the parameters of each test question in the system can be refined. As the volume of accumulated data gradually increases, the item parameters will become increasingly accurate, thereby enabling iterative optimization of the system. The purpose of the diagnosis is to provide feedback about each student’s learning status, primarily including the probability of each student mastering each attribute, and further generate the personalized learning pathways. Subsequently, it recommends remedial teaching resources based on the information of the diagnosis. The resulting interface of the assessment system is shown in Figure 7.

The interface depicted in Figure 7 displays the type, level, and quantity of each attribute mastered by an individual. Based on this information, recommendations tailored to students’ learning status can be generated and remedial teaching resources can be assigned.

### 3.3. Recommended System

The Recommended System is the core of the learning system. Testing and diagnostics are not the ultimate goals of the system. Instead, system focuses on solving problems after diagnosing them. The Recommended System facilitates personalized, targeted learning. Research has shown that student overload is primarily caused by excessive repetitive training (Ryan and Deci 2020). Acorrdingly, reducing unnecessary repetitive practice for students is an important way to improve learning efficiency. Meanwhile, another factor contributing to students’ learning difficulties is their lack of awareness of their own learning status (Dunlosky and Rawson 2015). The specific information contained in the diagnostic report provides the most direct basis for personalized learning.

In the Recommended System, the first step is to construct a learning resource repository—one of the more complex tasks in the development of learning systems. Learning resources need to be accurately coded by attribute, and this coding process needs to be done manually, particularly by mathematics experts. These experts are responsible for coding various types of resources, including micro-courses, micro-videos, practice problems, examples, and text explanations. Additionally, resources are uploaded at the management level, with simultaneous efforts to ensure effective compatibility of multi-format resources (e.g., videos, audios, and texts). The resource repository adapts an open, continuous iterative update model, allowing the sequential addition of new resources. However, each updated resource must align with the characteristics of the cognitive diagnostic assessment, that is, to meet the needs of the attribute test.

In the process of resource recommendation, we employed an attribute point-to-point similarity comparison approach. Based on the probability of students’ attribute mastery and the learning path they are currently following, we recommend targeted resources for students to learn. In the process of learning, we can retain the majority of the student’s learning information, which will be returned to the diagnostic information to become the next round of diagnostic data.

A computerized cognitive diagnosis-based adaptive learning system is a large cyclic cycle, where an adaptive cycle is formed through testing, diagnosis, and learning resource recommendation. Such cycles achieve the goal of adapting to different students, ultimately realizing personalized learning and enhancing students’ learning efficiency.

### 3.4. Case Analysis and Effectiveness Description

In order to further elucidate the operational process and effectiveness of the learning system, we will take J1 schools as a case study and provide an explanation in a sequential manner, covering the test system, diagnosis system and recommended system.

(1) Testing process

At J1 School, a total of 584 Grade 11 (Senior High Year 2) students from 12 classes participated in the “Equations and Inequalities” module assessment. Prior to the test, each student was assigned a unique username and password to ensure secure access. To guarantee the validity of the assessment, the school conducted it in a centralized manner with a standardized testing period of 90 min. Upon completion of the test, each student’s response data, along with the corresponding Q-matrix for the administered items, were systematically archived and forwarded to the assessment model for diagnostic scoring. The specific operational procedures and interface used by students during this process are delineated below.

**Figure 8:**
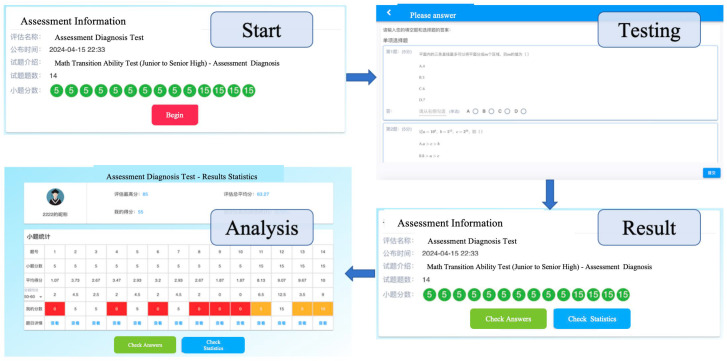
Main Operation Interface for Students. A red background indicates none, while an orange background indicates very little.

As shown in Figure 8, students can follow the system prompts to answer questions after logging into their account. Within 5 min of test completion, students can see the test results and associated performance statistics.

**Figure 9:**
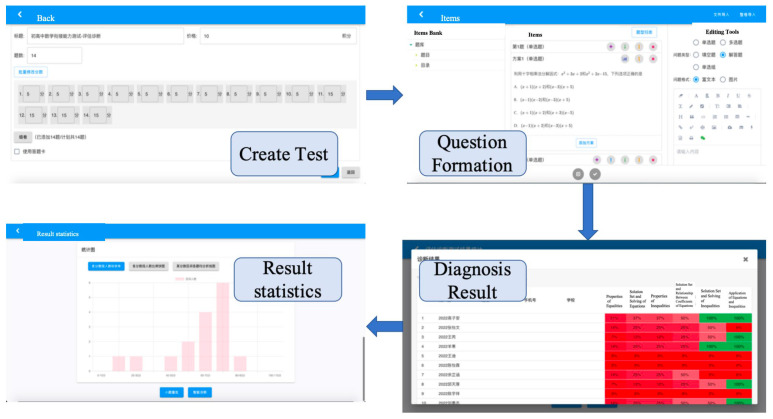
Main operation interface diagram of the teacher (administrator). A red background indicates little, while an green background indicates full score (100%).

In addition, teachers (administrators) will have different permissions in this system, such as creating tests, composing test questions, entering test questions, viewing each student’s results, and diagnostic analysis. The main operation interface is shown in Figure 9 below:

(2) Diagnostic process

Following the completion of the assessment, the system conducted an analysis of the response data collected from students in two classes at J1 School, integrating this newly acquired data with existing response data stored in the database. The system utilized a hybrid cognitive diagnostic modeling (CDM) approach, which entails applying distinct CDMs, customized to suit each individual test item, for the purpose of evaluation. For instance, the analysis of student J1-1-1 indicated a 100% probability of mastery for both the cognitive attributes “Solution Set and Solving of Inequalities” and the “Application of Equations and Inequalities” In contrast, the probabilities of mastery for other attributes were 21 percent for “Properties of Equations,” 37 percent for “Solution Set and Solving of Equations,” 37 percent for “Properties of Inequalities,” and 50 percent for “Relationship between Solution Set and Coefficients of Equations.” Similarly, corresponding mastery probabilities across different cognitive attributes were generated for all other students. This demonstrates that the diagnostic system developed in this study is capable of providing targeted diagnostic feedback on students’ mastery probabilities of specific cognitive attributes and generate personalized performance profiles for individual students.

(3) Referral process

Based on the diagnostic results of all participating students’ performance, the system can generate targeted recommendations and suggest appropriate instructional support resources. As previously mentioned, these support resources include micro-lessons, micro-videos, practice exercises, worked examples, and textual explanations. For instance, consider the student discussed earlier. Their 100% mastery probabilities for the cognitive attributes “Solution Set and Solving of Inequalities” and “Application of Equations and Inequalities” suggest that these students do not require redundant learning activities focused on these already mastered concepts. Conversely, their suboptimal mastery levels (as indicated by lower probabilities) for the attributes “Properties of Equations,” “Solution Set and Solving of Equations,” “Properties of Inequalities,” and “Relationship between Solution Set and Coefficients of Equations” prompt the system to provide targeted learning resources specifically designed to address these identified weaknesses. Such resources typically include relevant practice problems and explanatory videos.

(4) Effectiveness interview analysis

After a representative test of the schools participating in the test system trial, the research team selected representative teachers and students from the participating schools and conducted interviews. The interview content included the appropriateness of the test system, the quantity of test items, the mastery of cognitive attributes, the reasonableness of the probability analysis, and the quality of recommended learning resources. Below are some representative remarks from students:

*Student J1-1-3: During my study of the solid geometry part, I never had a good grasp of the problem of determining the parallel face of the surface. After completing the corresponding tests, the system pointed out that the probability of mastering this knowledge point was 35%, and pushed me a series of learning resources.*

*Student J1-4-11: The system has pushed me a lot of practice questions and explanations about the relationship between circles and the position of circles, which are easy to understand and I think it has helped me a lot.*

Some teachers also provided feedback on the system:

*Teacher J1-1: Based on the observation of the students’ answers and the diagnoses given, I think this system is very meritorious. Be able to give some valuable learning resources.*

*Teacher J1-4: This system may have the problem of a large number of questions in each push. On the whole, it has a great enlightening effect on teachers’ precision teaching.*

Based on the test results of these schools and the feedback from teachers and students, it was found that the system developed in this study can accurately diagnose students’ cognitive status and recommend teaching resources. Within the formative assessment component, the system continuously collected student response data during the learning process. Then, it employed the Q-matrix and hybrid CDM (Cognitive Diagnostic Modeling) to calculate real-time mastery probabilities for each cognitive attribute (e.g., 50% mastery of “Relationship between Solution Set and Coefficients of Equations”), dynamically generated personalized learning recommendations (e.g., avoiding redundant practice on mastered attributes), and intelligently recommended targeted remedial resources (e.g., micro-videos for weak attributes like “Properties of Equations”). This created an immediate “assessment-as-learning” feedback loop to support targeted remediation. Within the summative assessment component, upon module completion (e.g., post 90-min test), the system synthesized longitudinal data to generate a comprehensive cognitive diagnostic report. This report presented a mastery probability heatmap and proficiency level descriptors (e.g., Mastered/Partially Mastered/Not Mastered) for all target attributes (e.g., 11 skill modules), and supported student self-reflection and teacher instructional adjustments. Its reliability had been validated via test–retest consistency indices, thereby providing a scientific basis for academic achievement evaluation, teaching effectiveness assessment, and subsequent learning pathway planning. In general, the system is still in the process of continuous iteration, and such data feedback plays a crucial role in its ongoing improvement.

## 4. Discussion

With the rapid development of science and technology, the concept of adaptive online learning system has garnered growing attention in the field of education (Singh and Thurman 2019). Notably, accurately modeling students’ cognitive processes to improve their academic performance is a necessary requirement for a variety of educational systems (Griff and Matter 2013; Mavroudi et al. 2018; Tseng et al. 2008). This study aims to construct a cognitive diagnostic assessment system specifically tailored to the structural characteristics of China’s mathematics knowledge framework, and to leverage its diagnostic outcomes to drive students’ personalized learning. The core purpose of the system’s is to precisely characterize students’ mastery states of fine-grained cognitive attributes within specific mathematical knowledge modules (e.g., Equations and Inequalities) through the application of cognitive diagnostic models. In this way, it establishes a closed-loop “assessment → diagnosis → intervention” process, thereby facilitating a paradigm shift from uniform instruction to precision learning. This ability enables learners to select appropriate instructional activities, tasks and tools to support their development and progressively promote their understanding of increasingly complex concepts (Van Merriënboer and Sweller 2010).

A key advantage of cognitive diagnostic assessment (CDA) lies in its focus on students’ mental processes. Much existing research, while primarily concentrating on classifying cognitive attributes, has explored their interrelationships to a limited extent. The study prioritizes the systematic development of foundational cognitive models featuring hierarchical structures for knowledge modules. Specifically, it innovatively integrates the interpretive structural modeling (ISM) method with expert judgment to establish cognitive models for the 11 targeted knowledge modules. The ISM method, which relies on expert input to clarify logical relationships within a system, represents a subject logic-driven approach within the field of education (Tseng et al. 2008). In addition, in the development of an adaptive learning system for high school mathematics knowledge, this study innovatively explores processes such as system development and operation based on cognitive diagnostic theory. This effort may facilitate more effective application of cognitive diagnostic methods in education, especially in various disciplines of higher education. For instance, using cognitive diagnostics to assess knowledge at the level of higher education is a challenging yet highly innovative research direction that deserves further study. In conclusion, the integrated approach adopted in this study not only extends the application of interpretive structural modeling but also provides a clearer and more comprehensive way to construct cognitive diagnostic assessments. This successful attempt can also be generalized to the development of adaptive learning systems in other domains, thus enriching practical experience in the field.

In this study, we developed an adaptive online learning system for senior high school mathematics in China. Supported by cognitive diagnostic theory, the system functioned effectively in delivering accurate diagnostic assessments. However, the current knowledge base primarily relied on 11 relatively independent cognitive models and had not yet adequately modeled the dynamic interactions between different knowledge modules (Koedinger et al. 2012). In view of the high degree of intrinsic interconnectedness of mathematics itself (Huntington 1919), this neglect of interdependencies among knowledge components may limit the system’s diagnostic precision and instructional adaptability, particularly in the context of complex problem-solving and cross-topic knowledge transfer (Rittle-Johnson et al. 2015). A critical direction for the system’s future development is therefore to leverage data-driven approaches, such as mining large-scale learning behavior data to uncover knowledge association patterns (Baker and Yacef 2009; Siemens and Baker 2012) to identify and explicitly model the connections between these knowledge modules. This will enable the construction of a structured knowledge network that better aligns with the principles of mathematical cognition, thereby enriching the depth and breadth of the knowledge base.

Furthermore, factors influencing learning efficacy in senior high school mathematics extend far beyond the cognitive domain. Substantial research indicates that non-cognitive factors, including students’ emotional states (Pekrun 2006; Pekrun et al. 2017) and self-efficacy and belief in mathematics (Ayotola and Adedeji 2009; Zakariya 2022) during learning, exert a significant impact on problem-solving abilities, persistence, and overall academic performance (Richardson et al. 2012). Integrating these non-cognitive dimensions into the adaptive learning system enables a more holistic understanding of the learner’s state (Arroyo et al. 2014; D’Mello and Graesser 2012). By providing affective support, adjusting challenge levels, or offering motivational feedback (Ryan and Deci 2000), such system can psychologically optimize the learning experience, thereby enhancing student engagement and persistence.

In summary, the adaptive learning system developed in this study, after continuous integration of improvements across both cognitive and non-cognitive dimensions, holds significant potential for enhancing the quality of mathematics teaching and learning in high schools across China. Indeed, the current effectiveness of this system as well as the practical efficacy of its future enhanced iterations ultimately require validation rigorous large-scale empirical studies conducted within authentic Chinese high school educational contexts. Such studies should evaluate its specific contributions to elevating student academic achievement, learning motivation, and teacher instructional efficiency.

## 5. Research Limitations

This study introduces the development of an innovative adaptive learning system grounded in cognitive diagnostic assessment theory. Although this online learning system serves as a valuable tool for diagnosing student learning outcomes and providing feedback, creating accurate learning resources for senior high school mathematics remains challenging. Specifically, the development of the recommendation functionality relies on a structured resource repository; however, the current research has not yet systematically implemented metadata annotation (e.g., knowledge point relevance, difficulty level) for these learning resources. Consequently, our system currently lacks the capability to provide corresponding learning resources to students. Addressing this gap will constitute the paramount research direction for further enhancing the adaptive online learning system. In addition, the validity verification of the assessment tool of this system mainly relies on expert judgment, and variations in experts’ knowledge backgrounds and experience preferences may lead to significant differences in the structural model. Furthermore, this study did not conduct a long-term longitudinal study or randomized controlled trial, which to some extent limits the ability to assess its long-term impact on learning effectiveness. In conclusion, this study holds theoretical value in the construction of cognitive diagnostic models and the implementation of test diagnostic functions, but its limitations are mainly reflected in the lack of a closed-loop mechanism, insufficient dynamics, and limited empirical depth. Future work should focus on developing a resource recommendation module, optimizing the dynamic diagnosis algorithm, and verifying the system effectiveness through large-scale educational experiments. Furthermore, integrating generative AI and multimodal interaction technologies has the potential to further enhance the system’s intelligence and educational accessibility.

## Figures and Tables

**Table 1:** Knowledge modules involved in this study.

Compulsory Courses	Selective Compulsory Courses
Equations and inequalities The concept and properties of functions Basic solid geometry Basic properties of trigonometric functions Probability and Independence of Random Events Estimate the population using samples	Space vectors and solid geometry Discrete random variables and normal distribution Linear Regression and Independent Testing Circle and Equations Lines and Equations

**Table 2:** Cognitive attributes and coding of function concepts and properties.

Code	Cognitive Attributes	Content Description
**A1**	The concept of function	Understand the concept of functions; Ability to find the defined and valued domains of functions; Ability to discern the same function
**A2**	Representation of functions	Able to choose the appropriate method (image method, list method, analytic method) to represent functions according to different needs
**A3**	The monotonicity of functions and the applications	Understand the concept of functional monotonicity; Ability to judge and prove the monotonicity of functions; Ability to use monotonicity to find maximum and minimum values
**A4**	The parity of functions and the applications	Understand the concept of functional parity; Ability to judge and prove the parity of functions
**A5**	The application of the concept and properties of functions	Through concrete examples, students will learn about simple piecewise functions, and be able to solve problems by simply applying the concepts and properties of functions

**Table 3:** Adjacency matrix of the knowledge module of concept and properties of functions.

	A1	A2	A3	A4	A5
**A1**	0	1	0	0	0
**A2**	0	0	1	1	0
**A3**	0	0	0	0	1
**A4**	0	0	0	0	1
**A5**	0	0	0	0	0

**Table 4:** Final Q-matrix of the knowledge module of concept and properties of functions.

	A1	A2	A3	A4	A5
Item 1	1	0	0	0	0
Item 2	1	0	0	0	0
Item 3	1	0	0	0	0
Item 4	1	1	0	0	0
Item 5	1	1	0	0	0
Item 6	1	1	0	0	0
Item 7	1	1	1	0	0
Item 8	1	1	1	0	0
Item 9	1	1	1	0	0
Item 10	1	1	0	1	0
Item 11	1	1	0	1	0
Item 12	1	1	0	1	0
Item 13	1	1	1	1	1
Item 14	1	1	1	1	1
Item 15	1	1	1	1	1
Item 16	1	1	1	1	0
Item 17	1	1	1	1	0
Item 18	1	1	1	1	0

## Data Availability

The datasets generated and/or analyzed during the current study are available from the corresponding author upon reasonable request. The data are not publicly available due to ethical restrictions.
